# Telehealth abortion services via Women on Web in Kenya (2013–2019): a descriptive analysis of the characteristics and motivations of the care seekers

**DOI:** 10.1080/26410397.2025.2500828

**Published:** 2025-05-07

**Authors:** Mary Achieng Ouma, Anita Alaze, Kenneth Juma, Hazal Atay, Rebecca Gomperts, Céline Miani

**Affiliations:** a Doctoral student, Department of Epidemiology and International Public Health, School of Public Health, Bielefeld University, Bielefeld, Germany; b Senior Research Officer, African Population and Health Research Center (APHRC), Nairobi, Kenya; c Researcher, Women on Web International Foundation, Canada; Post-doctoral researcher, OFCE, Sciences Po Paris, Paris, France; d Director, Women on Waves, Netherlands; e Professor, Department of Epidemiology and International Public Health, School of Public Health, Bielefeld University, Universität Str. 25, Bielefeld 33615, Germany.

**Keywords:** self-managed abortion, telehealth, medical abortion, abortion stigma, Kenya, Women on Web

## Abstract

In Kenya, access to abortion is legally restricted and allowed under a limited set of conditions. Teleabortion service providers, such as Women on Web (WoW), provide safe and effective abortion care outside the formal health sector. This study explores the characteristics and motivations of individuals in Kenya who completed an online consultation on the WoW website in 2013–2019. We used anonymised data provided by WoW to describe participants’ characteristics (*n* = 857) and their motivations for accessing the WoW online consultation (*n* = 449, since this information was only available for those who filled out the questionnaire from December 2017). Participants’ median age was 23; 65.0% did not have children, and 80.9% had not had a previous abortion. Pregnancies were caused by failure (43.6%) or absence of contraceptive method (49.0%), or rape (6.0%). The most frequently reported reasons for accessing the online consultation were legal restrictions and abortion costs. Those were selected by about half the participants (respectively 235 and 222/449). Next came the wish to keep the abortion private or secret, which was selected by 34.5% and 26.0% of participants. Among more positively framed reasons, home comfort came first (23.6%), followed by wanting to deal with the abortion oneself (20.7%) and finding an abortion through WoW empowering (17.4%). Abortion-seekers turning to teleabortion services usually do so following failing or absent contraception and to get access to safe abortions, avoid stigma, and keep their privacy. Expansion of teleabortion services, within or outside formal healthcare services, could strengthen abortion-seekers’ autonomy and agency in Kenya.

## Plain language summary

In Kenya, access to abortion is legally restricted and allowed under a limited set of conditions. Teleabortion service providers, such as Women on Web (WoW), provide safe and effective abortion care outside the formal health sector: following an online consultation, they may provide abortion pills to persons experiencing an unwanted pregnancy. This study explores the characteristics of the individuals in Kenya who completed an online consultation on the WoW website in 2013–2019, as well as the reason why they decided to access the WoW website. We analysed anonymised data provided by WoW. Participants were, on average, 23 years old, 2/3 did not have children, and 80.9% did not have a previous abortion. Pregnancies were caused by failure (43.6%) or absence of contraceptive method (49.6%), or rape (6.0%). The most frequently reported reasons for accessing the online consultation were legal restrictions and abortion costs. About half the participants selected those. Next came the wish to keep the abortion private or secret, which was selected by, respectively, 34.5% and 26.0% of participants. Among more positively framed reasons, home comfort came first (23.6%), followed by wanting to take care of one’s abortion (20.7%) and finding an abortion through WoW empowering (17.4%). Abortion-seekers turning to teleabortion services usually do so following failing or absent contraception and with the intention of getting access to safe abortions, avoiding stigma, and keeping their privacy. Expansion of teleabortion services, within or outside formal healthcare services, could strengthen abortion-seekers’ autonomy and agency in Kenya.

## Résumé

Au Kenya, l’accès à l’avortement est restreint par la loi et autorisé dans un ensemble limité de cas. Les prestataires de services de télé-avortement, comme Women on Web (WoW), assurent des soins d’avortement sûrs et efficaces en dehors du secteur de la santé formel. Cette étude explore les caractéristiques et les motivations des personnes au Kenya qui ont effectué une consultation en ligne sur le site de WoW entre 2013 et 2019. Nous avons utilisé des données anonymisées fournies par WoW pour décrire les caractéristiques des participantes (*n* = 857) et leurs motivations pour accéder à la consultation en ligne de WoW (*n* = 449, car ces informations n’étaient disponibles que pour les personnes ayant rempli le questionnaire à partir de décembre 2017). L’âge médian des participantes était de 23 ans; 65.0% n’avaient pas d’enfants et 80,9% n’avaient jamais avorté. Les grossesses étaient dues à un échec de la contraception (43.6%) ou à son absence (49.0%), ou encore à un viol (6.0%). Les raisons les plus fréquemment invoquées pour accéder à la consultation en ligne étaient les restrictions juridiques et le coût de l’avortement. Ces raisons ont été sélectionnées par près de la moitié des participantes (respectivement 235 et 222/449). Venait ensuite le souhait de garder l’avortement privé ou secret, motif sélectionné par 34.5% et 26.0% des participantes. Parmi les raisons formulées de manière plus positive, le confort chez soi arrivait en tête (23.6%), suivi de la volonté de prendre en charge son avortement (20.7%) et l’idée qu’avorter par le biais de WoW donnait plus d'autonomie (17.4%). Les personnes voulant avorter qui ont recours aux services de télé-avortement le font généralement suite à un échec de la contraception ou une absence de méthode contraceptive et pour avoir accès à un avortement sûr, éviter la stigmatisation et protéger leur vie privée. L’expansion des services de télé-avortement, au sein ou en dehors des services de santé formels, pourrait renforcer l’autonomie et la capacité d’action des personnes souhaitant avorter au Kenya.

## Resumen

En Kenia, el acceso al aborto es restringido por las leyes y permitido bajo condiciones limitadas. Los prestadores de servicios de teleaborto, como *Women on Web* (WoW), proporcionan servicios de aborto seguros y eficaces fuera del sector salud formal. Este estudio explora las características y motivaciones de personas en Kenia que realizaron una consulta digital en el sitio web de WoW en 2013–2019. Utilizamos datos anonimizados proporcionados por WoW para describir las características de las participantes (*n* = 857) y sus motivaciones para acceder a la consulta digital de WoW (*n* = 449, dado que esta información estaba disponible solo para aquéllas que contestaron el cuestionario de diciembre de 2017). La edad media de las participantes era de 23; el 65.0% no tenía hijos y el 80.9% no había tenido un aborto anterior. Los embarazos fueron causados por la falla (43.6%) o ausencia de un método anticonceptivo (49.0%), o por violación (6.0%). Las razones relatadas con mayor frecuencia para acceder a la consulta digital fueron restricciones jurídicas y costos de aborto, ambas seleccionadas por aproximadamente la mitad de las participantes (respectivamente 235 y 222/449). La tercera razón más frecuente fue el deseo de mantener el aborto privado o en secreto, que fue seleccionada por el 34.5% y el 26.0% de las participantes. Entre las razones planteadas de manera más positiva, la primera fue la comodidad en el hogar (23.6%), seguida de querer hacerse cargo del aborto por sí misma (20.7%) y considerar empoderador tener un aborto por medio de WoW (17.4%). Las personas que buscan un aborto por medio de servicios de teleaborto generalmente lo hacen después de la falla o ausencia de anticoncepción y para obtener acceso a abortos seguros, evitar estigma y conservar su privacidad. La ampliación de los servicios de teleaborto, dentro o fuera de los servicios de salud formales, podría fortalecer la autonomía y agencia de las personas que buscan un aborto en Kenia.

## Introduction

### Access to abortion in Kenya

Over 98% of women in sub-Saharan Africa live in countries where abortion laws are restrictive.^1^ In Kenya, since 2010 and according to the constitution’s Article 26 (4), “abortion is not permitted unless, in the opinion of a trained health professional, there is need for emergency treatment, or the life or health of the mother is in danger, or if permitted by any other written law”.^2^ In 2019 rape was recognised as a legitimate ground for seeking abortions.^3^ These reforms marked gradual but critical progress toward the acknowledgment of reproductive healthcare, including abortion, as a fundamental right. The contradiction within the penal code persists though, which still criminalises abortion.^4^ This has led to wrongful arrests, persecutions, and harassment of women and healthcare providers and has fuelled fear and reluctance with regard to care-seeking and care provision.^5^ In 2012, the Ministry of Health withdrew standards and guidelines on safe abortion care, worsening the uncertainty on the legality of abortions,^6^ bringing to a halt training of providers, and contributing to unsafe abortions.^1^ Combined with the fact that many women and girls have vague or no knowledge of their sexual and reproductive health and rights,^7^ the uncertainty surrounding the legal status of abortion generates widespread abortion-related stigma.^8^

Given the restrictive nature of the legal and penal context, the vast majority of persons in need of abortion resort to unsafe abortion methods and procedures that ultimately lead to severe and life-threatening complications, as well as maternal near misses and deaths.^1^ In 2016, the government spent an estimated USD 4.4 million on treating complications from unsafe abortions in public health facilities.^9^ This inadequate access to care is also reflected in maternal mortality, whereby unsafe abortion accounts for approximately 35% of maternal mortality in Kenya,^10^ making it the leading cause of maternal mortality.^11^

### Medical abortions

With access to safe abortion being limited, particularly within the public health sector,^11^ people seeking safe abortions tend to rely on some private hospitals, clinics, and organisations, such as Marie Stopes Kenya^12^ and Safe2Choose.^13^ Those providers offer a range of services, including consultations for medical abortions through online platforms using the direct-to-clinic teleabortion model.^14^ Alternatively, persons seeking abortions can obtain abortion pills from pharmacies with a prescription from a physician. Barriers to accessing abortion medication include affordability and lack of availability of abortion medication, untrained providers at pharmacies, and stigma. Some of these challenges can be addressed through direct-to-consumer models,^14^ such as the services provided by Women on Web (WoW),^15^ a global telehealth abortion service that has been operating in Kenya since 2006. This model allows for safe, self-managed abortion* without any physical point of contact with formal services. Consequently, it promotes reproductive rights, autonomy, agency, and gender equality while challenging constraints imposed by restrictive gender norms and laws.^16^

In 2022, the World Health Organization (WHO) published updated guidelines on abortion. It introduced telemedicine for medical abortion as a mode of delivery with the potential to improve access to abortions safely and effectively.^17^ In the guidelines, telemedicine or telehealth^†^ for medical abortion entails the procurement of abortion pills by post, provided there is access to accurate information, quality-assured medicines, and support from a trained health worker if required. It is recommended up to 12 weeks of pregnancy. Those guidelines support the work of networks and organisations that have been providing abortifacients for years, sometimes decades, in contexts where abortion is illegal or access to abortion services is limited.^18^ Teleabortion services have been shown to address the unmet need for safe abortion services in a variety of settings.^19–21^

### Motivation for using teleabortion services

Research in other countries shows that the factors leading women to use telehealth abortion services are multifaceted and context-dependent (see for example,^22–25^). These can broadly be classified into three categories, namely empowerment, stigma, and access. Self-managed abortion can be emancipatory and empowering, allowing women to perform the abortion on their preferred terms in desired surroundings.^22^ Abortion stigma refers to the “negative attribute ascribed to women who seek to terminate a pregnancy that marks them, internally or externally, as inferior to ideals of womanhood” (p. 628).^26^ Stigma affects abortion-seekers, their supporters or families, as well as healthcare providers. Stigma is expressed mainly through exclusion, secrecy, denial, shame, guilt, silence, criminalisation, and negative attitudes of providers and is recognised as impeding access to safe abortion and post-abortion care. Last, access refers to the legal, socio-economic (e.g. access to education and job market), and health system-related context in which abortion-seekers navigate. This context is shaped by a country’s approach to reproductive rights and gender equality.^27^ Considering the ambiguity of the Kenyan law, we hypothesise that access issues and stigma may emerge as significant motivations for the uptake of telehealth abortion services. Still, we have no indication as to the magnitude of the empowerment dimension. To fill this gap, we analysed the characteristics and motivations of individuals in Kenya who completed an online consultation on the WoW website. The study findings can be a first step in identifying reasons why women in Kenya choose WoW, thus informing us about their needs and what matters to them in terms of abortion provision and providing leads about how to improve abortion services in Kenya in general.

## Methods

We analysed anonymised data from the WoW website available in Kenya. The WoW medical abortion service process begins with an online consultation, i.e. an online questionnaire that persons with an unwanted pregnancy have to fill. Respondents are then further referred to a doctor. If no contraindications are present, a medical abortifacient is mailed to them. The WoW consultation process has been described previously.^16^ The data used in this article relates to the initial online consultation and does not contain information on whether respondents were mailed abortion pills or did, in fact, have an abortion.

We aimed to include all cases available, from the opening of the Kenyan website (2006) to the COVID-19 pandemic. However, structural adjustments were made to the questionnaire over time, and some important variables were absent in the early years (e.g. obstetric history). We therefore ended up including online consultations from January 2013 to December 2019. The WoW questionnaire includes details on sociodemographic characteristics (age, residence), obstetric history, and information on current pregnancy (e.g. gestational age, cause of unintended pregnancy), as shown in Table 1. From December 2017, the questionnaire was updated to include a new question on motivations for accessing the WoW website, with 16 pre-specified answer options to select from (e.g. “it is hard for me to access abortion because of childcare”), as depicted in Figure 2. We chose not to include individuals who accessed the website during the COVID-19 pandemic (2020) onwards, as access to abortion services and the role of telehealth were likely affected by this public health crisis and the strain it placed on in-person healthcare services in general and abortion care in particular. Individuals who may not have considered teleabortion services before the pandemic could have sought WoW services because of those exceptional circumstances. At the time of data retrieval (2021), it was still too early to do an analysis of the changes due to the pandemic. A follow-up study researching this aspect would be a welcome addition to the field.

**Table 1:** Characteristics of the care seekers using the WoW telehealth abortion service, Kenya, 2013–2019 (*n *= 857)

Characteristics^a^		*n*	%
*Sociodemographic factors*			
**Age (in years)**	<20	113	13.19
	20–24	416	48.54
	25–29	194	22.64
	30–34	78	9.10
	35–39	41	4.78
	40–44	12	1.40
	>44	3	0.35
	*Missing*	*0*	*0*
**Region**^b^	Nairobi & Central	555	64.8
	Rift valley	143	16.7
	North Eastern & Coast	46	5.4
	Nyanza & Western	72	8.4
	Eastern	41	4.8
	*Missing*	*0*	*0*
**Hospital within ≤ 60-minute range**	Yes	789	92.1
	No	68	7.9
	*Missing*	*0*	*0*
*Obstetrics history*			
**Number of miscarriages**	0	755	88.1
	1	45	5.3
	2	7	0.8
	3	1	0.1
	*Missing*	*49*	*5*.*7*
**Number of children**	0	557	65.0
	1	164	19.1
	2	65	7.6
	>2	19	2.2
	*Missing*	*52*	*6*.*1*
**Number of abortions**	0	693	80.9
	1	105	12.3
	2	12	1.4
	>2	7	0.8
	*Missing*	*40*	*4*.*7*
*Characteristics of the pregnancy*			
**Support during the abortion process**	Somebody	779	90.9
	Nobody	78	9.1
	*Missing*	*0*	*0*
**Ultrasound**	No	610	71.2
	Yes	209	24.4
	*Missing*	*38*	*4*.*4*
**Cause of the unintended pregnancy**	No contraceptive used	420	49.0
	Contraceptive failure	374	43.6
	Rape	51	6.0
	*Missing*	*12*	*1*.*4*
**Gestation**	<7 weeks	731	85.3
	7–10 weeks	122	14.2
	>10 weeks	4	0.5
	*Missing*	*0*	*0*
**Reason for having an abortion**^+^	Cannot have a child at this point	411	48.0
	Financial burden	382	44.6
	School Commitment	371	43.3
	Young age	126	14.7
	Family complete	45	5.3
	Partner disagree	11	1.3
	Old age	9	1.1
	Illness	1	0.1
	*Missing*	*15*	*1*.*8*
Note: ^a^ Characteristics broken down for the earlier (2014–Nov. 2017) and latter (Dec. 2017–2019) sub-samples of care seekers are presented in Table A, supplementary material. They show little statistically significant differences in the sample over time. ^b^ We compiled the different locations the participants were from (42 counties) into five regions based not only on administrative locations but also as a reflection of distinct sociocultural patterns of the population and unique regional health profiles. ^+^Multiple answers possible.			

### Analysis

We presented descriptive statistics of the participants’ characteristics, using the categories available in the questionnaire except for the following: for age, we created categories (e.g. <20; 20–24); for the number of abortions and children, we grouped the highest values (>2); for the location, we attributed a region to each city (see Table 1 note). For the motivations for accessing the WoW online consultation, we presented the 16 pre-specified reasons for using WoW services and additionally sorted them into the three overarching categories described in the Introduction, namely empowerment, stigma, and access, based on the literature on self-managed abortion (e.g.^22,28^) and discussions within the authors’ team in relation to the Kenyan context. Those categories are often interdependent and reflect the main motivations for accessing abortion medication outside of formal healthcare services. We used IBM SPSS Statistics 28.

### Ethics statement

The lead authors (MO, AA and CM) signed a confidentiality agreement with WoW who shared the anonymised data. Care seekers accessing the WoW website gave their consent for their data to be shared in an anonymised form for research purposes before starting the online consultation process. Following a change of policy with regard to anonymised secondary data, this study was approved retrospectively by the ethics committee of Bielefeld University (decision n.2025-46 on 13th March 2025).

### Positionality statement

As public health researchers grounded in a personal and professional commitment to reproductive justice, we approach this study with the conviction that access to safe, legal, and stigma-free abortion is a fundamental component of health equity and human rights. As funder of and researcher for Women on Web, RG and HA have been involved in efforts to enable abortion access for all. We recognise the importance of transparency in our positionality, and we see our roles not as neutral observers, but as engaged participants working to support and amplify reproductive autonomy globally.

### Patient involvement

No patients were involved in this study’s design, conduct, or reporting.

## Results

**Figure 1.**
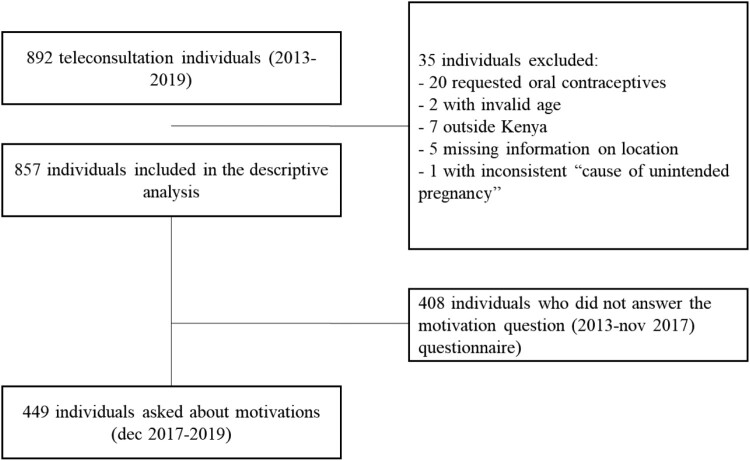
Teleconsultation participants flow chart

We included 857 individuals in the descriptive analyses. Among them, 449 filled the questionnaire from December 2017 onwards, and were therefore asked about their motivations for accessing the WoW website. (Figure 1).

The median age of participants was 23 ± 5.3 (Table 1). The majority (64.8%) came from the Nairobi and Central region. In terms of reproductive history, 65.0% did not have children, and the majority did not have a previous abortion (80.9%) or miscarriage (88.1%). Regarding the current pregnancy, most participants reported being pregnant for less than 7 weeks (85.3%), and about 25% underwent an ultrasound confirming the pregnancy. In most cases, pregnancy was caused by the absence of contraception (49%) or failure of contraceptive method (43.6%). Another cause of pregnancy was rape, which was reported by 51 of the participants (6.0%). Regarding the reason for having an abortion, school commitment (43.3%), financial burden (44.6%), and “cannot have a child at this point” (48.0%) were the most reported reasons.

**Figure 2.**
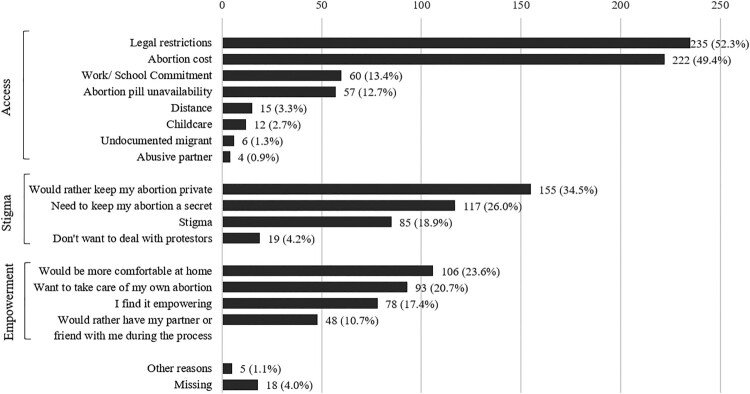
Motivations for accessing the WoW online consultation, Kenya, 2017–2019 (*n *= 449). Note: The exact question reads “What are the main reasons why you are requesting an abortion through Women on Web?” Answers are pre-specified options that women can select. The question is optional, which explains the missing data (*n *= 18). Multiple responses are allowed, and respondents tended to give multiple answers, so that the total response exceeds the number of respondents (*n *= 449).

For the persons who accessed the WoW online consultation from December 2017 (n = 449), we have insight as to why they chose to seek medical abortion care specifically through WoW. Those reasons are presented in Figure 2, grouped in the three categories “access”, “stigma” and “empowerment”. Reasons related to access were the most frequently reported reasons, with legal restrictions and abortion costs selected by about half the participants (respectively 235 and 222/449, or 52.3% and 49.4%). Stigma reasons came second, with the wish to keep the abortion private or secret being selected by respectively 155 and 117 (or 34.5% and 26%) of respondents. Among the empowerment reasons, home comfort came first (106/449 or 23.6%), followed by wanting to take care of one’s own abortion (93/449 or 20.7%) and finding an abortion through WoW services empowering (78/449 or 17.4%).

## Discussion

This is the first study to investigate the characteristics and motivations of persons accessing telehealth abortion services in Kenya. In terms of reasons for seeking an abortion, we found that the timing of pregnancy and the size of one’s family were some of the main reasons for seeking an abortion. Those are well-documented reasons in the literature, as is continuing one’s education (“school commitment”). Our study is indeed consistent with the evidence on in-person abortion services in Kenya^29^ and highlights once more the central role abortion plays in family planning and in giving (young) women an additional opportunity to remain in control of their reproductive trajectories and on the path to their aspirational life goals. This opportunity is particularly important in a context like Kenya, where contraceptive-related stigma and the culturally ingrained desire for high fertility and having many children in families thwart access to and utilisation of contraception and reproductive health services.^30^ In our sample, in 49.0% of the cases, the unwanted pregnancy was due to an absence of contraception, and in 43.7% of cases to failing contraception. Those two issues call for a multi-faceted approach to sexual and reproductive health and rights. Interventions aiming at addressing persisting unmet contraception needs and contraception-related stigma, in particular among the youth, have to be intertwined with any action or programme seeking to improve access to abortion care.

It is also worth noting that 1 in 4 participants in the online consultation had their pregnancy confirmed through an ultrasound; this suggests that those persons had access to reproductive health services but still explored the possibility of having an abortion through WoW. This may be yet another indication of the limited capacity of the formal health care system to respond to the needs and expectations of abortion-seekers – something that could be explored further in a study on care trajectories of persons seeking an abortion.

Access-related issues were the most reported motivations for accessing the WoW teleconsultation. They included legal restrictions, immigrant status, perceived cost, unavailability of services, challenges due to distance, commitment to education and work, and childcare. Among them, legal restrictions and the cost of abortion services were selected by about half of the participants. This reflects the unmet need for safe and financially accessible abortion services and the risks that accompany using them. While abortion is authorised under some circumstances and acknowledged as commonplace in the Kenyan community (in 2012, it was estimated that 49% of all pregnancies in Kenya were unintended, and 41% of unintended pregnancies ended in abortion),^5^ it is still viewed in society as objectionable and a deviation from social norms, which attracts various forms of ostracisation and discrimination.^31^ Notably, these perspectives are rooted in religious, moral, ethical, and socio-cultural beliefs,^31^ which may not align with what the law in principle allows. Similarly, although the availability of abortion pills increased since they were termed essential drugs in 2016 by law, their provision remains a challenge. The dispatch of abortion medication is hindered by the fact that providers in pharmacies fear persecution,^31^ leading them to sell the drugs at varying prices (≈US $58 and higher).^31–33^ Online telehealth services such as those of WoW provide an alternative. In support of abortion care, a High Court of Malindi ruling affirmed in 2022 that abortion care is a fundamental right under the Constitution of Kenya and that arbitrary arrests and prosecution of patients and healthcare providers for seeking or offering such services is illegal.^34^ The effects of this ruling will have to be investigated, but it constitutes a step in bridging the gap between the law and its (lack of) implementation.

Participants in the online consultation also opted for telehealth to circumnavigate stigma-related concerns encompassing secrecy, privacy, and anti-abortion protests. This is in line with the findings of several qualitative studies on abortion in Kenya (e.g.^29,31^) and, again, points to the importance of cultural context even in settings where abortion law can be considered relatively liberal. The online services offered by organisations such as WoW are inherently discreet and allow a short-term solution to secrecy/privacy needs. However, those services should be complemented with public health action that actively fights abortion-related stigma in communities. As pointed out by a recent scoping review,^35^ the evidence on interventions to reduce abortion-related stigma is scarce, and more means should be invested in designing and evaluating interventions that tackle stigma at different levels (e.g. internalised and intrapersonal, but also structural stigma).

Last, with regards to empowerment, we observed that many women selected affirmative reasons for using WoW services. First among them came the notion of the comfort of the home, taking care of the abortion oneself, and feeling empowered. This resonates with the broader literature on medical abortion, which underlines how wishes for control or self-management can lead individuals to choose medical abortion over surgical abortion in settings where both methods are available.^36^ Overall, the motivations for accessing the WoW online consultation in Kenya are comparable to reasons for seeking an abortion with WoW across countries with different, sometimes less restrictive, legal abortion contexts.^21,22,28,37,38^ They suggest that investing in dematerialised and flexible teleabortion services would be valued by abortion-seekers and could help address the current unmet needs in terms of access to safe abortion services in Kenya.

By leveraging technology through their digital platform, WoW empowers individuals – especially those in underserved or legally constrained areas – to exercise their right to bodily autonomy and make informed choices about their reproductive futures. Medical abortion through telehealth aligns with a rights-based understanding of sexual and reproductive health, including the right and access to safe abortion care.^39^ Services that provide confidential and evidence-based care, free from discriminatory practices, contribute to bridging critical gaps in healthcare systems.^40^ For many in Kenya, access to WoW could be life-saving, reducing unsafe abortion practices and mitigating maternal mortality. WoW’s model exemplifies how technology can be harnessed to uphold and expand sexual and reproductive rights, fostering a more just and equitable healthcare landscape.^41^

### Limitations

Our findings are generalisable to all care seekers using the WoW website in Kenya during the study period. However, at the scale of the country, this cross-sectional sample is relatively small and not representative of the general population of abortion-seekers in Kenya: WoW website users may differ from users of other (telehealth or formal healthcare) services in terms of characteristics and motivations. Some important individual-level factors are missing from our study, such as religion and marital status. Yet, some important characteristics are comparable to other sources: for example, in a representative sample of the Kenyan population, about 16% of women seeking post-abortion services for an induced abortion reported to have had (at least) a previous induced abortion (vs. 14.5% in our sample).^42^ Last, we could only analyse motivations for using WoW services for a subset of the participants starting when this variable became available. An analysis of the evolution of characteristics and motivations of the care seekers over time, through the latest health reforms, and through the COVID-19 pandemic and beyond, was not possible within the scope of this article. A follow-up study exploring potential changes would bring additional understanding of the contemporary role of telehealth abortion services in Kenya.

## Conclusion

This paper offers new insights into the characteristics and motivations of persons seeking abortion through the telehealth service WoW in Kenya. Findings reveal how stigma and access issues, but also empowerment, redirect women to seek self-managed abortions outside of formal health services. Building on the latest guidelines from the WHO, there could be an opportunity for Kenyan health services to support the development of teleabortion services through a range of private organisations, and in the public health sector.

## Author contributions

*Conceptualisation: MO, AA, CM. Formal analysis: MO, AA, CM. Methodology: MO, AA, CM. Supervision: CM. Visualisation: CM, MO. Writing – original draft: MO, AA, KJ, HA, RG, CM. Writing – review & editing: MO, AA, KJ, HA, RG, CM. Data curation: HA. Project administration: HA.*

## Data Availability

WoW data can be made available for research purposes upon reasonable request.

## Footnotes

* Self-managed abortion refers to “any action that is taken to end a pregnancy outside of the formal healthcare system, and could include self-sourcing medications”. Verma N, Grossman D. Self-Managed Abortion in the United States. Curr Obstet Gynecol Rep. 2023;12(2):70–75.

† A term preferred by feminist networks and online abortion pill providers, considering the fact that they promote self-managed abortion and challenge the traditional medicalisation of abortion.

## Appendix

**Table A1:** Characteristics of the care seekers using the WoW telehealth abortion service, Kenya, 2013–2019 (*n* = 857), broken down between two time periods: 2014–November 2017 (*n* = 408) and December 2017–2019 (*n* = 449)

		Total		2014–November 2017		December 2017–2019		*P*-value
		*N* = 857	%	*N* = 408	%	*N* = 449	%	
*Sociodemographic factors*								
**Age**								0.175
	<20	113	13.19	53	13.0	60	13.4	
	20–24	416	48.54	185	45.3	231	51.5	
	25–29	194	22.64	99	24.3	95	21.2	
	30–34	78	9.10	39	9.6	39	8.7	
	35–39	41	4.78	25	6.1	16	3.6	
	40–44	12	1.40	7	1.7	5	1.1	
	>44	3	0.35	*0*	*0*	3	0.7	
	*Missing*	*0*	*0*	*0*	*0*	*0*	*0*	* *
**Region** ^a^	* *	* *	* *	* *	* *	* *	* *	0.170
	Nairobi & Central	555	64.8	278	68.14	277	61.7	
	Rift valley	143	16.7	63	15.44	80	17.8	
	North Eastern & Coast	46	5.4	21	5.15	25	5.6	
	Nyanza & Western	72	8.4	33	8.09	39	8.7	
	Eastern	41	4.8	13	3.19	28	6.2	
	*Missing*	*0*	*0*	*0*	*0*	*0*	*0*	* *
**Hospital within ≤ 60-minute range**	* *	* *	* *	* *	* *	* *	* *	<0.001
	Yes	789	92.1	391	95.8	398	88.6	
	No	68	7.9	17	4.2	51	11.4	
	*Missing*	*0*	*0*	*0*	*0*	*0*	*0*	* *
*Obstetrics history*								
**Number of miscarriages**								0.190
	0	755	88.1	368	90.2	387	86.2	
	1	45	5.3	24	5.9	21	4.7	
	2	7	0.8	1	0.3	6	1.3	
	3	1	0.1	1	0.3	0	0	
	*Missing*	*49*	*5*.*7*	*14*	*3*.*4*	*35*	*7*.*8*	* *
**Number of children**	* *	* *	* *	* *	* *	* *	* *	0.937
	0	557	65.0	277	67.9	280	62.4	
	1	164	19.1	77	18.9	87	19.4	
	2	65	7.6	32	7.8	33	7.4	
	>2	19	2.2	9	2.2	10	2.2	
	*Missing*	*52*	*6*.*1*	*13*	*3*.*2*	*39*	*8*.*7*	* *
**Number of abortions**	* *	* *	* *	* *	* *	* *	* *	0.650
	0	693	80.9	337	82.6	356	79.3	
	1	105	12.3	54	13.2	51	11.4	
	2	12	1.4	4	1.0	8	1.8	
	>2	7	0.8	4	1.0	3	0.7	
	*Missing*	*40*	*4*.*7*	*9*	*2*.*2*	*31*	*6*.*9*	* *
*Characteristics of the pregnancy*								
**Support during the abortion process**								0.222
	Somebody	779	90.9	376	92.2	403	89.8	
	Nobody	78	9.1	32	7.8	46	10.2	
	*Missing*	*0*	*0*	*0*	*0*	*0*	*0*	* *
**Ultrasound**	* *	* *	* *	* *	* *	* *	* *	<0.001
	No	610	71.2	237	58.1	373	83.1	
	Yes	209	24.4	144	35.3	65	14.5	
	*Missing*	*38*	*4*.*4*	*27*	*6*.*6*	*11*	*2*.*5*	* *
**Cause of the unintended pregnancy**	* *	* *	* *	* *	* *	* *	* *	0.647
	No contraceptive used	420	49.0	208	51.0	212	47.2	
	Contraceptive failure	374	43.6	178	43.6	29	6.5	
	Rape	51	6.0	22	5.4	196	43.7	
	*Missing*	*12*	*1*.*4*	*0*	*0*	*12*	*2*.*7*	* *
**Gestation**	* *	* *	* *	* *	* *	* *	* *	<0.001
	<7 weeks	731	85.3	311	76.2	420	93.5	
	7–10 weeks	122	14.2	97	23.8	25	5.6	
	>10 weeks	4	0.5	*0*	*0*	4	0.9	
	*Missing*	*0*	*0*	*0*	*0*	*0*	*0*	* *
**Reason for having an abortion** ^+^	Cannot have a child at this point	411	48.0	206	50.5	205	45.7	0.252
	Financial burden	382	44.6	191	46.8	191	42.5	0.315
	School Commitment	371	43.3	182	44.6	189	42.1	0.622
	Young age	126	14.7	68	16.7	58	12.9	0.153
	Family complete	45	5.3	26	6.4	19	4.2	0.182
	Partner disagree	11	1.3			11	2.5	0.001
	Old age	9	1.1	5	1.2	4	0.9	0.653
	Illness	1	0.1	0	0	1	0.2	0.335
	*Missing*	*15*	*1*.*8*	*3*	*0*.*7*	*12*	*2*.*7*	* *
Note: ^a^ We compiled the different locations the participants were from (42 counties) into five regions based not only on administrative locations but also as a reflection of distinct sociocultural patterns of the population and unique regional health profiles. +Multiple answers possible.								
One can see that the characteristics of the care seekers in the two time periods are very similar. The only variables that show a statistically significant difference (*p* < 0.001) between the samples are: *ultrasound, gestational age, proximity of a hospital* and “*partner disagree”* in *reason for having an abortion*. Some of those suggest potential changes in the healthcare environment (e.g. *ultrasound*, *hospital*), but the absence of the *reason for using WoW* in the earlier sample does not allow to see if those changing parameters could have had an impact on the motivations of the care seekers.								
